# Effect of Protease Supplementation in Diets with or Without Copper Sulfate and Formaldehyde on the Standardized Digestibility of Amino Acids in Broiler Chickens

**DOI:** 10.3390/ani15071059

**Published:** 2025-04-05

**Authors:** Ingryd Palloma Teodósio da Nobrega, Levy do Vale Teixeira, Vitor Barbosa Fascina, Letícia Cardoso Bittencourt

**Affiliations:** DSM-Firmenich, Innovation and Applied Science, Mairinque 18120-000, SP, Brazillevy.teixeira@dsm-firmenich.com (L.d.V.T.); vitor.fascina@dsm-firmenich.com (V.B.F.)

**Keywords:** amino acid digestibility, broiler chickens, copper sulfate, formaldehyde, protease

## Abstract

With the growing demand for sustainable and cost-effective poultry production, optimizing nutrient utilization is crucial. Exogenous proteases enhance protein digestibility, reduce nitrogen excretion, and improve feed efficiency, but their interaction with additives like copper sulfate (CuSO_4_) and formaldehyde remains unclear. This study evaluated the combined effects of protease with these additives on amino acid digestibility, performance, and bone quality in broilers. A total of 500 broilers were assigned to seven dietary treatments, each replicated 10 times (six birds per cage) over 21 days (14 days of adaptation and 7 days of evaluation). Treatments included a reference diet (RD), RD + CuSO_4_ (600 g/ton, 150 mg Cu/kg), RD + formaldehyde (2000 g/ton), with or without protease supplementation (30,000 NFP/kg), and a protein-free diet. Protease improved protein digestibility (2.50%) and amino acid digestibility (essential: 2.64%; non-essential: 2.52%) in diets with CuSO_4_ or formaldehyde (*p* < 0.05). CuSO_4_ alone had no effect, but its combination with protease significantly enhanced crude protein digestibility (4.63%). Formaldehyde reduced amino acid digestibility, but protease mitigated this negative effect (−4.68% vs. −1.81%). Protease also improved feed conversion and bone strength. These findings demonstrate that protease combined with CuSO_4_ or formaldehyde optimizes nutrient digestibility, enhances broiler performance, and supports sustainable poultry production.

## Simple Summary

Efficient and sustainable poultry production depends on maximizing nutrient utilization. This study examined how a protease enzyme interacts with feed additives like copper sulfate and formaldehyde in broiler diets. A total of 500 broilers were observed for 21 days under different feeding conditions. Results showed that protease improved protein and amino acid digestion, essential for poultry nutrition. When combined with copper sulfate, protein digestibility significantly increased. Formaldehyde negatively affected amino acid absorption, but protease helped reduce this impact. Additionally, broilers fed with protease had better feed conversion (meaning they utilized feed more efficiently) and stronger bones. These findings suggest that using protease alongside these additives can enhance nutrient absorption, improve broiler performance, and support a more sustainable poultry production process by reducing waste and maximizing feed efficiency.

## 1. Introduction

With advancements in poultry nutrition, strategies to optimize feed efficiency and reduce protein costs, one of the most expensive components of diets, are essential [1]. Exogenous proteases have emerged as a promising solution to enhance protein digestibility and provide essential amino acids, enabling more cost-effective and efficient diets without compromising animal performance [2]. Additionally, they reduce nitrogen excretion, mitigating environmental impacts such as water pollution and greenhouse gas emissions [3]. These enzymes also optimize feed conversion and improve gut health, meeting the demands of sustainable and competitive production [4,5,6]. Over the years, protease technology has advanced significantly, now reaching its fourth generation, with improvements in stability, specificity, and resistance to gastrointestinal conditions. These advancements have increased their versatility in feed formulation and expanded their applicability across diverse dietary compositions. Given their long-standing use, proteases have been extensively studied in the literature, with a well-established body of research validating their efficacy [7,8,9]. Beyond maximizing nutrient utilization, ensuring the microbiological safety and quality of animal feed is a critical aspect of poultry production, as contaminated feed can pose significant health risks to poultry and, by extension, to human consumers of animal-derived products. In this context, the use of formaldehyde in feed, although banned in the European Union since 2018 due to occupational safety concerns, is permitted, regulated, and widely used in some regions as an additive in animal feed to mitigate feed contamination by pathogens [10,11,12]. Alongside these technologies, other additives targeting gut health have been widely adopted in poultry production. Copper sulfate (CuSO_4_), for example, when used at levels higher than those recommended to meet broiler chickens’ nutritional needs, has stood out for its antimicrobial properties and growth-promoting effects. It aids in controlling intestinal pathogens, improving gut health, and enhancing feed efficiency, which translates into weight gain, better feed conversion, and reduced reliance on antibiotic growth promoters [13,14,15,16]. All these technologies are extensively utilized and well-documented regarding their benefits and efficacy when used individually. However, uncertainties remain about their potential synergies or antagonisms when combined in broiler diets, given the reactive or sensitive nature of these additives. Recent advancements in protein engineering have enabled the development of more robust and thermostable enzymes capable of withstanding adverse conditions during feed processing and storage [17]. However, due to the chemical characteristics of enzymes, caution is required when adding ingredients to feed to avoid interactions or damage that might compromise enzyme functionality [18]. Similarly, concerns exist about formaldehyde’s potential impact on nutrient bioavailability [12,19], as it reacts with proteins to form methylol groups, Schiff bases, and methylene bridges, making them less susceptible to digestive enzymes [20]. Additionally, limited studies suggest formaldehyde-based products may reduce phytase activity in feed [21,22]. Likewise, CuSO_4_, due to its weak ionic bonds and high solubility, can be highly reactive, potentially damaging the intestinal mucosa and muscular layer, oxidizing feed nutrients, and reducing phytase efficacy [23,24,25,26,27,28]. Nutrient digestibility is a critical factor in broiler nutrition, directly influencing performance and health. Enzyme supplementation, particularly with proteases, has been widely studied for its ability to improve protein and amino acid digestibility. However, protease efficacy may be affected by additives such as CuSO_4_ and formaldehyde, which are often used for their antimicrobial and preservative properties. Understanding how these additives interact with proteases is essential to optimizing feed formulations and maximizing nutritional benefits. Based on this, our research aimed to evaluate the impact of protease supplementation on the standardized ileal digestibility of amino acids, as well as on performance and bone quality, when combined with coadjuvant additives such as CuSO_4_ and formaldehyde. The study seeks to clarify whether these additive combinations offer a synergistic effect or if antagonisms compromise nutritional efficacy and poultry health.

## 2. Materials and Methods

### 2.1. Animals, Facilities, and Experimental Design

The study was conducted at the ANC—Animal Nutrition Center (dsm-firmenich) in Mairinque, São Paulo, Brazil. A total of 500 one-day-old male Cobb MV broiler chicks were used, vaccinated at the hatchery against Marek’s, Gumboro, and Newcastle diseases. At the start of the experiment (14 days old), the birds were individually weighed (489 g ± 4.31 g) and allocated to stainless steel metabolic cages equipped with nipple drinkers and trough feeders in a climate-controlled facility. The temperature and lighting program followed the guidelines of the Cobb manual. Birds had ad libitum access to water and feed. The experimental design included seven treatments with 10 replicates, each comprising six birds, totaling 70 experimental units in a completely randomized design. The experiment lasted 21 days.

### 2.2. Experimental Diets

A three-phase feeding program was used: pre-starter (1–7 days), starter I (8–14 days), and starter II (15–21 days). All birds received a standard diet from day 1 to 14, followed by experimental diets from day 15 to 21. Experimental diets were corn and soybean meal-based, with or without on-top protease supplementation (30,000 NFP/kg feed) without nutritional matrix valuation, coadjuvant additives (formaldehyde-based product and CuSO_4_), and a protein-free diet. The experimental design, dietary compositions, and the protein-free diet formulation are presented in Table 1, Table 2 and Table 3. The diets were analyzed using near-infrared spectroscopy (NIR) for mineral matter (MM), ether extract (EE), crude fiber (CF), moisture (MO), phytate, and starch content. Nitrogen content was determined using the Dumas method (LECO) and multiplied by 6.25 to calculate crude protein (CP) levels [29]. Phytase and protease concentrations in the diets were analyzed using the RapidLab method (DSM RapidLab Hiphos and ProAct360 powered by MICT Assay Kit—MagnaBioAnalytics, LLC, San Diego, CA, USA). The average results are described in Table 4.

### 2.3. Performance Data

At the start and end of the digestibility evaluation phase (15–21 days), birds and feed were weighed to measure weight gain and feed intake. Mortality was recorded daily to calculate the mortality rate, correct feed intake, and determine feed conversion efficiency.

### 2.4. Amino Acid Digestibility

At 21 days of age, all birds were slaughtered to collect ileal digesta. The ileal content was freeze-dried, ground, and analyzed for acid-insoluble ash [29], dry matter [30], nitrogen [29], and total amino acids [31], as well as the experimental diets. Apparent and standardized ileal amino acid digestibility were calculated following the methodology proposed by Ravindran et al. [32].

Apparent Ileal Digestibility Coefficient (AIDC)

(1) $$AIDC\;\left(\%\right)=\frac{\left[\left(\frac{AA}{AIA}\right)\;diet-\left(\frac{AA}{AIA}\right)\;digesta\right]}{\left(\frac{AA}{AIA}\right)\;diet}\times 100$$

(AA/AIA) diet = ratio of amino acid to AIA in the diet;

(AA/AIA) digesta = ratio of amino acid to AIA in the ileal digesta.

Standardized Ileal Digestibility Coefficient (SIDC)

(2) $$SIDC\left(\%\right)=AIDC\left(\%\right)+(\frac{BEAAL}{AA\;diet})$$

BEAAL (g/kg of dry matter intake) = basal endogenous amino acid loss;

AA diet (g/kg of dry matter) = amino acid concentration in the feed.

### 2.5. Tibia Analysis

At 21 days of age, the tibias were collected from the same birds slaughtered for ileal collection and analyzed for bone strength and tibia ash percentage. To determine bone strength or bone-breaking strength (maximum breaking force), the tibiotarsus was subjected to a three-point bending test at a constant deformation rate for viscoelastic material using a texture analyzer (TA-XT2i Model, Stable MicroSystems Ltd., Godalming, UK). The parameters used were a speed of 1 mm/sec, a force of 10 g, and a stress of 12 mm. For bone ash analysis, the adherent tissue and joint capsules of the right tibiotarsus were removed, and the bones were dried in an oven at 105 °C for 24 h. They were then placed in petroleum ether for 24 h for defatting. Afterward, the bones were dried again in an oven at 105 °C for 24 h and subsequently ashed in a muffle furnace at 600 °C for 10 h to determine the ash percentage [33].

### 2.6. Statistical Analysis

The data were analyzed as a completely randomized 3 × 2 factorial design, with 3 diet treatments (reference, CuSO_4_, and formaldehyde) and 2 levels of protease supplementation (with and without the enzyme). Data were subjected to a two-way ANOVA using the GLM procedure of SAS^®^ Studio (SAS Institute Inc., Cary, NC, USA). When significant (*p* < 0.05), means were compared using Tukey’s test.

## 3. Results

### 3.1. Protein and Amino Acid Digestibility

There was an interaction between protease supplementation and the coadjuvant additives on protein and amino acid (AA) digestibility, except for cystine (*p* < 0.05) (Table 5 and Table 6). Protease, in combination with coadjuvant additives, significantly improved crude protein digestibility (2.50%), essential AA digestibility (2.64%; except valine), and non-essential AA digestibility (2.52%; except cystine) (*p* < 0.05), particularly when combined with CuSO_4_. The use of CuSO_4_ alone in the basal diet did not alter crude protein or AA digestibility. However, when combined with protease, it resulted in a 4.63% improvement in crude protein digestibility and a 1.76% to 5.28% increase in AA digestibility (*p* < 0.05) (Table 5 and Table 6). In this study, formaldehyde alone in the basal diet reduced crude protein and AA digestibility. Formaldehyde treatments decreased AA and protein digestibility by 4.50% and 4.68%, respectively (*p* < 0.05) (Table 5 and Table 6). Protease supplementation in the diet mitigated this negative effect, reducing the AA digestibility loss from −4.49% (without protease) to −1.81% (with protease).

### 3.2. Productive Performance

There was no interaction between protease supplementation and coadjuvant additives on broiler performance variables (*p* > 0.05) (Table 7). However, protease supplementation improved the feed conversion ratio by 2.32% in broilers raised in metabolic cages (*p* < 0.05). In this study, CuSO_4_ addition to the diet did not significantly improve broiler performance compared to the reference diet. However, the feed conversion ratio was significantly better in birds fed diets with CuSO_4_ compared to those fed formaldehyde-treated diets (*p* < 0.05), while the performance of birds on the basal or formaldehyde diets was similar (*p* > 0.05).

### 3.3. Bone Strength and Quality

Similarly to productive performance, there was no interaction between protease supplementation and coadjuvant additives on bone quality in broilers (*p* > 0.05) (Table 8). However, protease supplementation had a significant effect on bone strength and ash percentage (*p* < 0.05). Results showed that protease inclusion increased tibial bone strength and ash percentage by 9.72% and 2.65%, respectively (*p* < 0.05). In contrast, neither formaldehyde nor CuSO_4_ addition affected bone quality parameters (*p* > 0.05).

## 4. Discussion

Protease supplementation in broiler diets improves protein and amino acid (AA) digestibility by catalyzing the breakdown of peptide bonds, converting complex proteins into smaller peptides and free AAs, thereby facilitating absorption in the gastrointestinal tract. Cowieson and Roos [33], in a meta-analysis evaluating the effect of a specific protease on apparent ileal AA digestibility in poultry and swine diets, observed an average improvement of +3.74% (SE 1.1%; *p* < 0.001). Similarly, Lee et al. [9] reported an improvement of +1.6 ± 0.3% for most AAs in protease-supplemented poultry and swine diets (*p* < 0.05). Although copper alone did not significantly improve AA digestibility, Kirchgessner et al. [34] suggested that it may enhance pepsin activation, facilitating greater protein absorption. Additionally, studies show that copper, in various forms, provides notable benefits for intestinal functionality, potentially optimizing nutrient utilization. These benefits include altering intestinal morphology and physiology [35], inhibiting pathogens such as Escherichia coli [36,37], coliforms [38], and *Clostridium* [39], reducing lesions caused by Eimeria [40], and stimulating Lactobacillus populations in the gut [41]. Considering the positive impact of proteases on protein digestibility and their similarity to copper in improving gut functionality [42,43], the complementary effects of these additives likely contributed synergistically to the observed improvements in AA digestibility. The effect of formaldehyde on AA digestibility remains controversial. Feye [44] reported increased methionine digestibility with the inclusion of 2.5 kg/ton of formaldehyde-based products. However, other studies found no significant effect on protein digestibility [45,46]. Conversely, Williams et al. [12] and Ochoa et al. [47] found that formaldehyde-based products could reduce AA availability in swine diets. Similarly to its use in mitigating protein–formaldehyde crosslinking in immunohistochemistry and vaccine development [48,49], protease likely reduced the negative effects of formaldehyde in this study. Although the crosslinking induced by formaldehyde increases protein resistance to protease action, most modifications may actually enhance protein susceptibility to enzymatic proteolysis and accelerate the rate of proteolytic degradation [49]. This change is possibly due to the greater accessibility of proteases to cleavage sites and structural modifications of the proteins. Regarding zootechnical performance, the improvement observed in this study is likely due to enhanced AA digestibility promoted by the action of protease. This occurs because more efficient nutrient utilization is essential to meet the metabolic demands of rapidly growing animals, providing the necessary substrates for protein synthesis, muscle development, and efficient feed conversion. Recent studies, such as Stefanello et al. [50], reported improvements of 3.4% and 2.5% in weight gain and feed conversion, respectively, in broiler diets with reduced AA levels supplemented with the protease in question. Similarly, Vieira et al. [51] observed a reduction in the feed conversion ratio of animals fed diets containing different levels of the same protease, regardless of the dosages evaluated. Additionally, Cowieson et al. [52] found that adding a novel exogenous protease to broiler diets improved weight gain (+7.3%) and reduced the feed conversion ratio (−4%), even in diets already containing two other feed enzymes (phytase and xylanase). Freitas et al. [53] observed that protease supplementation improved feed efficiency regardless of the protein and energy levels tested in the experiment. In their meta-analysis, Lee et al. [9] found feed conversion improvements of −0.92% for the various proteases studied (*p* < 0.01). Although CuSO_4_ was not able to provide significant improvements in broiler performance parameters compared to the reference diet, other studies have highlighted CuSO_4_’s effects on improving broiler performance [13,54]. Conversely, formaldehyde inclusion has often been associated with negative performance outcomes in monogastric diets [10,11,47]. Regarding bone strength, studies evaluating the isolated effect of protease on this parameter are scarce. However, when evaluating the effect of protease, either in combination with or without phytogenics, on the bone quality of broilers, Hafeez et al. [55] observed an increase in tibia weight in animals supplemented with protease. According to the author, this finding may be correlated with greater weight gain and digestibility indices observed in birds fed the studied enzyme. It is suggested that the superior bone quality parameters observed in animals supplemented with protease in this study are due to the enzyme’s extra-proteolytic effects, as documented by Cowieson and Roos [8]. These effects could include greater calcium and phosphorus digestibility, as demonstrated in the results of Farrokhi et al. [56] and Olukosi et al. [57]. Since the nutrients in feed ingredients are incorporated within a complex matrix, it is natural for feed enzymes to exert broader impacts beyond their target nutrients. In this context, proteases also play a significant role in improving the digestibility of non-protein nutrients. This effect may be attributed to significant changes in the structure of the nutrient matrix following proteolysis or other factors, such as endogenous secretion, gut health, and active nutrient transport [16]. Furthermore, although the studies by Farrokhi et al. [56] and Olukosi et al. [57] do not fully clarify how protease improved calcium and phosphorus utilization, it is likely that, similar to phytase, which increases AA availability by preventing and breaking protein–phytate complexes [58], protease, by focusing on breaking the protein portions of these complexes, may facilitate the action of phytase present in the diet. This improvement may enhance phytate hydrolysis, making it more accessible. Consequently, the antinutritional effects of phytate could be reduced, and the bioavailability of phosphorus and calcium—essential minerals for bone formation—could increase. For a more robust and in-depth analysis of the potential synergistic effects between phytase and protease, it is recommended that future studies include treatments with phytase alone, as well as combined treatments with both enzymes. This approach will allow a clear comparison of the individual and combined impacts of these enzymes on nutrient digestibility, particularly phosphorus and calcium.

## 5. Conclusions

The results of this study indicate that protease supplementation, when combined with CuSO_4_ or formaldehyde, significantly improves protein and AA digestibility in broilers. Although CuSO_4_ alone did not affect crude protein and AA digestibility, its combination with protease resulted in substantial improvements. On the other hand, formaldehyde alone reduced digestibility, but the addition of protease mitigated this negative effect. Furthermore, protease supplementation also improved the feed conversion ratio and bone strength in broilers, indicating additional benefits for performance and health. From a practical standpoint, these findings suggest that protease can be effectively incorporated into broiler diets alongside CuSO_4_ and formaldehyde without compromising its efficacy, providing greater flexibility in feed formulation and enhancing nutrient utilization. However, further research is needed to fully elucidate the mechanisms underlying these interactions and to explore additional aspects beyond nutrient utilization, zootechnical performance, and bone mineralization.

## Figures and Tables

**Table 1:** Description of dietary treatments.

Treatments Identification	Description
1	Basal Diet (BD) + Protease (Prot.) *^1^
2	BD + Prot. + CuSO_4_ *^2^
3	BD + Prot. + Formaldehyde *^3^
4	BD *^1^
5	BD + CuSO_4_ *^2^
6	BD + Formaldehyde *^3^
7	Protein-Free Diet

*^1^ Inclusion of 50 g/ton protease (30,000 NFP), on-top. *^2^ Copper Sulfate Pentahydrate 600 g/ton of feed, 150 mg of Cu/kg of feed. *^3^ Formaldehyde-based commercial product, 2000 g/ton of feed, 700 g formaldehyde/ton of feed.

**Table 2:** Nutritional composition of the experimental diets (15 to 21 days of age).

Ingredients	Quantity (kg)
Corn 7.5%	564
Soybean meal 45%	370
Soybean Oil	25.00
Inert *	2.00
Limestone 40%	9.40
Dicalcium phosphate 27/19.5	6.30
Salt	4.80
DL- Methionine	3.10
L-Lysine	1.90
L-Threonine	0.80
Choline Chloride 60%	0.60
HiPhorius 40 (1000 FYT)	0.025
Vitamin premix **	13.00
Mineral premix ***	0.50
Insoluble acid ash (Celite^TM^)	10.00
Total	1.000
Nutritional Levels (%)	
Metabolizable energy (kcal/kg)	2.976
Crude protein %	21.28
Crude Fiber %	2.93
Lysine (A-Dig)	1.180
Methionine (A-Dig)	0.599
Amino acids (A-Dig)	0.874
Threonine (A-Dig)	0.767
Tryptophane (A-Dig)	0.228
Arginine (A-Dig)	1.316
Isoleucine (A-Dig)	0.801
Valine (A-Dig)	0.885
Ether extract	5.161
Calcium	0.860
Total Phosphorus	0.670
Phosphorus Disp. Poultry	0.430
Sodium	0.200

* Inclusion of 600 g/ton of Penta Hydrated Copper Sulfate; 2000 g/ton of formaldehyde-based product and 50 g/ton of protease (30,000 NFP), replacing inert according to dietary treatments. ** Vitamin A (min) 11,000,000 IU, Vitamin D3 (min) 4,000,000 IU, Vitamin E (min) 55,000 IU, Vitamin K3 (min) 3000 mg, Vitamin B1 (min) 2300 mg, Vitamin B2 (min) 7000 mg, Pantothenic acid (min) 12 g, Vitamin B6 (min) 4000 mg, Vitamin B12 (min) 25,000 mcg, Niacin acid (min) 60 g, Folic acid (min) 2000 mg, Biotin (min) 250 mg, Selenium (min) 300 mg. *** Iron (min) 100 g, Copper (min) 20 g, Manganese (min) 130 g, Zinc (min) 130 g, Iodine (min) 2000 mg.

**Table 3:** Composition of the protein-free diet (15 to 21 days of age).

Ingredients	Quantity (kg)
Corn starch	201
Dextrose	638
Soybean oil	50.0
Monocalcium phosphate 22.7/16	19.0
Limestone 40	13.0
Corn cob	50.0
Vitamin premix	1.30
Mineral premix	0.500
Choline Chloride	2.50
Insoluble acid ash (Celite^TM^)	10.0
Potassium carbonate	2.60
Potassium chloride	2.90
Sodium bicarbonate	7.50
Magnesium oxide	2.00
Total	1000

**Table 4:** Analyzed composition of experimental diets provided during the period from 15 to 21 days of age regarding phytate, ether extract (EE), crude fiber (CF), mineral matter (MM), moisture, crude protein (CP), phytase, and protease.

Treatments	Phytate (%)	EE (%)	CF (%)	MM (%)	Moisture (%)	CP (%)	Phytase (FYT/kg)	Protease (NFP/kg)
Basal Diet (BD) + Protease (Prot.)	0.240	6.22	2.36	5.73	11.1	22.9	483	34,500
BD + Prot. + CuSO_4_	0.250	6.22	2.50	5.86	11.1	21.9	612	31,400
BD + Prot. + Formaldehyde	0.240	6.41	2.44	5.82	11.2	22.1	812	40,200
BD	0.250	6.28	2.33	5.57	10.3	21.7	781	<9000
BD + CuSO_4_	0.260	6.28	2.34	5.82	10.4	22.5	806	<9000
BD + Formaldehyde	0.230	6.36	2.57	5.87	11.1	22.1	527	<9000
Protein-Free Diet	-	-	-	-	-	0.52	<100	<9000

**Table 5:** Standardized ileal digestibility coefficients of protein and essential amino acids of broiler chickens at 21 days of age.

Protease	Co-Adjuvant Additive					*p*-Value		
	Basal Diet	CuSO_4_	Formaldehyde	Average	SEM	Protease	Additive Co-Adjuvant	Interaction
-----Crude protein-----								
With	86.3 ^B^ *	90.3 ^Aa^	85.3 ^Ba^	87.3	0.553	0.0344	<0.0001	0.0018
Without	87.7 ^A^	87.7 ^Ab^	83.6 ^Bb^	86.3				
Average	87.0	89.0	84.4					
-----Lysine-----								
With	93.5 ^B^	96.1 ^A^	92.4 ^Ba^	94.0	0.434	0.0138	<0.0001	0.0001
Without	94.7 ^A^	95.1 ^A^	89.4 ^Bb^	93.1				
Average	94.1	95.6	90.9					
----- Methionine -----								
With	96.8 ^B^	98.5 ^Aa^	97.3 ^Ba^	97.5	0.268	0.0284	0.0001	0.0042
Without	97.4 ^A^	97.4 ^Ab^	96.3 ^Bb^	97.0				
Average	97.1	98.0	96.8					
-----Threonine-----								
With	87.9 ^B^	92.1 ^Aa^	88.1 ^B^	89.4	0.678	0.0786	0.0009	0.0067
Without	89.0	88.7 ^b^	87.4	88.4				
Average	88.5	90.4	87.7					
-----Valine-----								
With	89.2 ^Bb^	93.2 ^A^	89.1 ^B^	90.5	0.619	0.3614	<0.0001	0.0061
Without	91.2 ^Aa^	91.4 ^A^	87.4 ^B^	90.0				
Average	90.2	92.3	88.3					
-----Isoleucine-----								
With	90.0 ^B^	93.5 ^A^	89.4 ^Ba^	91.0	0.584	0.2434	<0.0001	0.0097
Without	91.7 ^A^	92.1 ^A^	87.5 ^Bb^	90.4				
Average	90.8	92.8	88.5					
-----Arginine-----								
With	94.6 ^B^	96.7 ^Aa^	93.0 ^Ca^	94.8	0.390	0.0003	<0.0001	<0.0001
Without	95.6 ^A^	95.5 ^Ab^	89.5 ^Bb^	93.5				
Average	95.1	96.1	91.3					
-----Phenylalanine-----								
With	91.3 ^B^	94.5 ^Aa^	90.5 ^Ba^	92.1	0.475	0.0131	<0.0001	0.0022
Without	92.4 ^A^	92.7 ^Ab^	88.1 ^Bb^	91.1				
Average	91.9	93.6	89.3					
-----Leucine-----								
With	90.6 ^B^	94.0 ^Aa^	90.2 ^Ba^	91.6	0.512	0.0311	<0.0001	0.0058
Without	91.7 ^A^	91.9 ^Ab^	88.3 ^Bb^	90.7				
Average	91.2	93.0	89.3					
-----Histidine-----								
With	89.8 ^B^	91.9 ^Aa^	89.0 ^Ba^	90.2	0.590	0.0015	<0.0001	0.0007
Without	91.0 ^A^	89.1 ^Ab^	85.6 ^Bb^	88.6				
Average	90.4	90.5	87.3					

* A,B uppercase letters on the same line differ from each other regarding the effect of the coadjuvant additive (*p* < 0.05). a,b lowercase letters in the same column differ from each other regarding the effect of protease (*p* < 0.05).

**Table 6:** Standardized ileal digestibility of protein and non-essential amino acids of broiler chickens at 21 days of age.

Protease	Co-Adjuvant Additive					*p*-Value Additive		
	Basal Diet	CuSO_4_	Formaldehyde	Average	SEM	Protease	Co-Adjuvant	Interaction
----- Alanine -----								
With	89.7 ^B^ *	93.4 ^Aa^	89.9 ^B^	91.0	0.590	0.3888	<0.0001	0.0206
Without	91.0	91.3 ^b^	89.4	90.6				
Average	90.4	92.4	89.6					
----- Aspartic Acid -----								
With	89.6 ^B^	92.3 ^Aa^	88.3 ^Ba^	90.1	0.499	0.0030	<0.0001	0.0031
Without	90.4 ^A^	90.4 ^Ab^	85.6 ^Bb^	88.8				
Average	90.0	91.4	86.9					
----- Cystine -----								
With	82.0	88.2	82.1	84.1 b	1.093	0.0031	<0.0001	0.3666
Without	83.0	91.6	86.2	86.9 a				
Average	82.5 ^B^	89.9 ^A^	84.1 ^B^					
----- Glycine-----								
With	87.1 ^Bb^	91.7 ^Aa^	86.7 ^B^	88.5	0.676	0.1739	<0.0001	0.0037
Without	89.0 ^Aa^	89.1 ^Ab^	85.0 ^B^	87.7				
Average	88.0	90.4	85.9					
----- Glutamic acid -----								
With	93.4 ^B^	95.4 ^Aa^	91.6 ^Ca^	93.5	0.387	0.0016	<0.0001	0.0003
Without	94.1 ^A^	94.2 ^Ab^	88.9 ^Bb^	92.4				
Average	93.8	94.8	90.2					
----- Proline -----								
With	89.5 ^B^	92.4 ^Aa^	88.2 ^B^	90.0	0.554	0.1322	<0.0001	0.0242
Without	90.6 ^A^	90.7 ^Ab^	86.7 ^B^	89.3				
Average	90.1	91.5	87.5					
----- Serine -----								
With	89.7 ^B^	93.2 ^Aa^	89.1 ^B^	90.7	0.628	0.0140	<0.0001	0.0085
Without	90.6 ^A^	90.0 ^Ab^	87.5 ^B^	89.4				
Average	90.2	91.6	88.3					
----- Tyrosine -----								
With	90.8 ^B^	94.2 ^Aa^	90.6 ^Ba^	91.9	0.639	0.0531	<0.0001	0.0446
Without	91.7 ^A^	92.4 ^Ab^	88.4 ^Bb^	90.8				
Average	91.3	93.3	89.5					

* A,B uppercase letters on the same line differ from each other regarding the effect of the coadjuvant additive (*p* < 0.05). a,b lowercase letters in the same column differ from each other regarding the effect of protease (*p* < 0.05).

**Table 7:** Average final weight (AFW, kg), average feed intake (AFI, kg/bird), average weight gain (AWG, kg/bird), and feed conversion ratio (FCR, kg/kg) of broiler chickens from 15 to 21 days of age.

Treatments		Variables			
Protease	Processing	AFW, kg	AFI, kg/Bird	AWG, kg/Bird	FCR, kg/kg
WITH	Basal diet	0.951	0.506	0.461	1.098
WITH	CuSO_4_	0.956	0.495	0.461	1.073
WITH	Formaldehyde	0.938	0.499	0.450	1.109
WITHOUT	Basal diet	0.938	0.499	0.452	1.105
WITHOUT	CuSO_4_	0.957	0.511	0.461	1.106
WITHOUT	Formaldehyde	0.930	0.523	0.458	1.146
	CV (%) ^1^	5.20	6.97	7.53	3.42
Main Effects					
Protease	WITH	0.948	0.500	0.458	1.093
	WITHOUT	0.941	0.511	0.457	1.119
Processing	Basal diet	0.944	0.503	0.457	1.101 ^ab^
	CuSO_4_	0.957	0.503	0.462	1.089 ^b^
	Formaldehyde	0.934	0.511	0.454	1.127 ^a^
*p*-value	Coadjuvant additives	0.3422	0.6860	0.7897	0.0082
	Protease	0.6091	0.2158	0.9851	0.0100
	Interaction	0.9060	0.3606	0.7357	0.4134

^1^ Coefficient of variation. Means with different letters in the same column differ significantly according to Tukey’s test (*p* < 0.05).

**Table 8:** Bone strength and percentage of ash in the tibia of broilers at 21 days of age.

Treatments		Variables	
Protease	Processing	Bone Strength (kgf/cm^2^)	Ash (%)
WITH	Basal Diet	32.94	35.51
WITH	CuSO_4_	36.59	35.94
WITH	Formaldehyde	31.02	35.25
WITHOUT	Basal Diet	29.75	34.55
WITHOUT	CuSO_4_	31.72	35.49
WITHOUT	Formaldehyde	30.20	33.94
	CV (%) ^1^	17.81	4.59
Main effects			
Protease	WITH	33.52 ^A^	35.57 ^A^
	WITHOUT	30.55 ^B^	34.65 ^B^
Processing	Basal diet	31.33	35.03
	CuSO_4_	34.15	35.71
	Formaldehyde	30.61	34.59
*p*-value	Coadjuvant additives	0.1261	0.0931
	Protease	0.0494	0.0318
	Interaction	0.5331	0.6939

^1^ Coefficient of variation. Means with different letters in the same column differ significantly according to Tukey’s test (*p* < 0.05).

## Data Availability

The data presented in this study are available from the corresponding author upon reasonable request.

## References

[1] Beski S.S.M., Swick R.A., Iji P.A. (2015). Specialized protein products in broiler chicken nutrition: A review. Anim. Nutr..

[2] Adeola O., Cowieson A.J. (2011). Board-Invited Review: Opportunities and challenges in using exogenous enzymes to improve nonruminant animal production. J. Anim. Sci..

[3] Leinonen I., Williams A.G. (2015). Effects of dietary protease on nitrogen emissions from broiler production: A holistic comparison using Life Cycle Assessment. Anim. Feed Sci. Technol..

[4] McCafferty K.W., Morgan N.K., Cowieson A.J., Choct M., Moss A.F. (2022). Varying apparent metabolizable energy concentrations and protease supplementation affected broiler performance and jejunal and ileal nutrient digestibility from 1 to 35 d of age. Poult. Sci..

[5] Xu X., Wang H.L., Pan L., Ma X.K., Tian Q.Y., Xu Y.T., Long S.L., Zhang Z.H., Piao S.L. (2017). Effects of coated proteases on the performance, nutrient retention, gut morphology and carcass traits of broilers fed corn or sorghum-based diets supplemented with soybean meal. Anim. Feed Sci. Technol..

[6] Cowieson A.J., Abdollahi M.R., Zaefarian F., Pappenberger G., Ravindran V. (2018). The effect of a mono-component exogenous protease and graded concentrations of ascorbic acid on the performance, nutrient digestibility, and intestinal architecture of broiler chickens. Anim. Feed Sci. Technol..

[7] Cowieson A.J., Roos F.F. (2014). Bioefficacy of a mono-component protease in the diets of pigs and poultry: A meta-analysis of effect on ileal amino acid digestibility. J. Appl. Anim. Nutr..

[8] Cowieson A.J., Roos F.F. (2016). Toward optimal value creation through the application of exogenous mono-component protease in the diets of non-ruminants. Anim. Feed Sci. Technol..

[9] Lee S.A., Bedford M.R., Walk C.L. (2018). Meta-analysis: Explicit value of mono-component proteases in monogastric diets. Poult. Sci..

[10] EFSA FEEDAP Panel (EFSA Panel on Additives and Products or Substances used in Animal Feed) (2014). Scientific Opinion on the safety and efficacy of formaldehyde for all animal species based on a dossier submitted by Regal BV. EFSA J..

[11] Ricke S., Richardson K., Dittoe D. (2019). Formaldehydes in feed and their potential interaction with the poultry gastrointestinal tract microbial community: A review. Front. Vet. Sci..

[12] Williams H.E., Cochrane R.A., Woodworth J.C., DeRouchey J.M., Dritz S.S., Tokach M.D., Jones C.K., Fernando S.C., Burkey T.E., Li Y.S. (2018). The effects of dietary supplementation of formaldehyde and crystalline amino acids on gut microbial composition of nursery pigs. Sci. Rep..

[13] Arias V.J., Koutsos E.A. (2006). Effects of copper source and level in intestinal physiology and growth of broiler chickens. Poult. Sci..

[14] Burnell T.W., Cromwell G.L., Stahly T.S. (1988). Effects of dried whey and copper sulfate on the growth responses to organic acid in diets for weanling pigs. J. Anim. Sci..

[15] Hill G.M., Cromwell G.L., Crenshaw T.D., Dove C.R., Ewan R.C., Knabe D.A., Lewis A.J., Libal G.W., Mahan D.C., Shurson G.C. (2000). Growth promotion effects and plasma changes from feeding high dietary concentrations of zinc and copper to weanling pigs (regional study). J. Anim. Sci..

[16] Skrivan M., Skrivanova V., Marounek M., Tumova E., Wolf J. (2000). Influence of dietary fat source and copper supplementation on broiler performance, fatty acid profile of meat and depot fat, and on cholesterol content in meat. Br. Poult. Sci..

[17] Rigoldi F., Donini S., Redaelli A., Parisini E., Gautieri A. (2018). Review: Engineering of thermostable enzymes for industrial applications. AIP Bioeng..

[18] Bedford M.R., Partridge G.G. (2010). Enzymes in Farm Animal Nutrition.

[19] Campbell J.M., Crenshaw J.D., Polo J., Mellick D., Bienhoff M., Stein H.H. (2018). Impact of formaldehyde-treated pig feed containing spray-dried plasma on weaned pig growth performance. J. Anim. Sci..

[20] Metz B., Kersten G., Hoogerhout P., Brugghe H.F., Timmermans H., Jong A.D., Meiring H.D., Hove J.T., Hennink W.E., Crommelin D.J. (2004). Identification of formaldehyde-induced modifications in proteins. J. Biol. Chem..

[21] Santos T.T., Gomes G.A., Walk C.L., Freitas B.V., Araujo L.F. (2013). Effect of formaldehyde inclusion on phytase efficiency in broilers. J. Appl. Poult. Res..

[22] Sheehan N., Bedford M.R., Partridge G.G. (2010). Analysis of enzymes, principles, and problems: Developments in enzyme analysis. Enzymes in Farm Animal Nutrition.

[23] Banks K.M., Thompson K.L., Jaynes P., Applegate T.J. (2004). The effects of copper on the efficacy of phytase, growth, and phosphorus retention in broiler chicks. Poult. Sci..

[24] Chiou P.W.S., Chen C.L., Chen K.L., Wu C.P. (1999). Effect of high dietary copper on the morphology of the gastrointestinal tract in broiler chickens. Asian-Australas. J. Anim. Sci..

[25] Hamdi M., Solà D., Franco R., Durosoy S., Roméo A., Pérez J.F. (2018). Including copper sulfate or dicopper oxide in the diet of broiler chickens affects performance and copper content in the liver. Anim. Feed Sci. Technol..

[26] Lu L., Wang R.L., Zhang Z.J., Steward F.A., Luo X., Liu B. (2010). Effect of dietary supplementation with copper sulfate or tribasic copper chloride on the growth performance, liver copper concentrations of broilers, and stabilities of vitamin E and phytase in feeds. Biol. Trace Elem. Res..

[27] Miles R.D., Keefe S.F.O., Henry P.R., Ammerman C.B., Luo X.G. (1998). The effect of dietary supplementation with copper sulfate or tribasic copper chloride on broiler performance, relative copper bioavailability, and dietary prooxidant activity. Poult. Sci..

[28] Pang Y., Applegate T.J. (2006). Effects of copper source and concentration on in vitro phytate phosphorus hydrolysis by phytase. J. Agric. Food Chem..

[29] (2023). Compêndio Brasileiro de Alimentação Animal. Métodos Analíticos.

[30] (2004). AOAC Official Methods of Analysis.

[31] White J.A., Hart R.J., Fry J.C. (1986). An evaluation of the Waters Pico-Tag system for the amino-acid analysis of food materials. J. Autom. Chem..

[32] Ravindran V., Adeola O., Rodehutscord M., Kluth H., van der Klis J.D., van Eerden E., Helmbrecht A. (2017). Determination of ileal digestibility of amino acids in raw materials for broiler chickens—Results of collaborative studies and assay recommendations. Anim. Feed Sci. Technol..

[33] Latimer G.W. (2023). AOAC Official Methods of Analysis of AOAC International.

[34] Kirchgessner M., Beyer M.G., Steinhart H. (1976). Activation of pepsin (EC 3.4.4.1) by heavy-metal ions including a contribution to the mode of action of copper sulfate in pig nutrition. Br. J. Nutr..

[35] Di Giancamillo A., Rossi R., Martino P.A., Aidos L., Maghin F., Domeneghini C., Corino C. (2018). Copper sulfate forms in piglet diets: Microbiota, intestinal morphology, and enteric nervous system glial cells. Anim. Sci. J..

[36] Danilova T.A., Danilina G.A., Adzhieva A.A., Vostrova E.I., Zhukhovitskii V.G., Cheknev S.B. (2020). Inhibitory effect of copper and zinc ions on the growth of Streptococcus pyogenes and Escherichia coli biofilms. Bull. Exp. Biol. Med..

[37] Xia M.S., Hu C.H., Xu Z.R. (2004). Effects of copper-bearing montmorillonite on growth performance, digestive enzyme activities, and intestinal microflora and morphology of male broilers. Poult. Sci..

[38] Højberg O., Canibe N., Poulsen H.D., Hedemann M.S., Jensen B.B. (2005). Influence of dietary zinc oxide and copper sulfate on the gastrointestinal ecosystem in newly weaned piglets. Appl. Environ. Microbiol..

[39] Santos T., Teng P.Y., Yadav S., Castro F., Koch R., Craig S., Chen C., Fuller A., Pazdro R., Sartori J. (2020). Effects of inorganic Zn and Cu supplementation on gut health in broiler chickens challenged with *Eimeria* spp. Front. Vet. Sci..

[40] Chen J., Yan F., Kuttappan V.A., Wedekind K., Vázquez-Añón M., Hancock D. (2023). Effects of bis-chelated copper on growth performance and gut health in broiler chickens subject to coccidiosis vaccination or coccidia challenge. Front. Physiol..

[41] Peng C.C., Yan J.Y., Dong B., Zhu L., Tian Y.Y., Gong L.M. (2017). Effects of graded levels of cupric citrate on growth performance, antioxidant status, serum lipid metabolites and immunity, and tissue residues of trace elements in weaned pigs. Asian-Australas. J. Anim. Sci..

[42] Cardinal K., Moraes M., Andretta I., Schirmann G., Belote B., Barrios M., Santin E., Machado A., Ribeiro L. (2019). Growth performance and intestinal health of broilers fed a standard or low-protein diet with the addition of a protease. Rev. Bras. Zootec..

[43] Perez-Palencia J.Y., Samuel R.S., Levesque C.L. (2021). Supplementation of protease to low amino acid diets containing superdose levels of phytase for wean-to-finish pigs: Effects on performance, postweaning intestinal health, and carcass characteristics. Transl. Anim. Sci..

[44] Feye K.M., Dittoe D.K., Jendza J.A., Caldas-Cueva J.P., Mallmann B.A., Booher B., Tellez-Isaias G., Owens C.M., Kidd M.T., Ricke S.C. (2021). A comparison of formic acid or monoglycerides to formaldehyde on production efficiency, nutrient absorption, and meat yield and quality of Cobb 700 broilers. Poult. Sci..

[45] Jones M.K., Richardson K.E., Starkey C.W., Dale N.M., Davis A.J. (2018). Impact of extended heat treatment on the amino acid digestibility and TMEn content of a formaldehyde-treated diet. J. Appl. Poult. Res..

[46] Yakhkeshi S., Rahimi S., Naseri K.G. (2011). The effects of comparison of herbal extracts, antibiotic, probiotic, and organic acid on serum lipids, immune response, GIT microbial population, intestinal morphology, and performance of broilers. J. Med. Plants.

[47] Ochoa L., Harrell R.J., Graham A., Bienhoff M., Kremer B., Loughmiller J.A., Greiner L. (2017). Effect of feeding formaldehyde-treated feed to pigs throughout the growing period on amino acid utilization from synthetic lysine or protein sources. J. Anim. Sci..

[48] Kouchmeshky A., McCaffery P. (2020). Use of fixatives for immunohistochemistry and their application for detection of retinoic acid synthesizing enzymes in the central nervous system. Methods Enzymol..

[49] Michiels T.J.M., Meiring H.D., Jiskoot W., Kersten G.F.A., Metz B. (2020). Formaldehyde treatment of proteins enhances proteolytic degradation by the endo-lysosomal protease cathepsin S. Sci. Rep..

[50] Stefanello C., Dalmoro Y.K., Rosa D.P., Teixeira L., Sorbara J.O.B., Cowieson A.J., Faruk M.U. (2024). Effects of dietary digestible amino acids and a novel exogenous protease on growth performance of broilers. Heliyon.

[51] Vieira S.L., Freitas C.R., Horn R.M., Favero A., Kindlein L., Sorbara J.O.B., Umar-Faruk M. (2022). Growth performance and nutrient digestibility of broiler chickens as affected by a novel protease. Front. Anim. Sci..

[52] Cowieson A.J., Smith A., Sorbara J.O.B., Pappenberger G., Olukosi O.A. (2019). Efficacy of a mono-component exogenous protease in the presence of a high concentration of exogenous phytase on growth performance of broiler chickens. J. Appl. Poult. Res..

[53] Freitas D.M., Vieira S.L., Angel C.R., Favero A., Maiorka A. (2011). Performance and nutrient utilization of broilers fed diets supplemented with a novel mono-component protease. J. Appl. Poult. Res..

[54] Samanta B., Biswas A., Ghosh P.R. (2011). Effects of dietary copper supplementation on production performance and plasma biochemical parameters in broiler chickens. Br. Poult. Sci..

[55] Hafeez A., Iqbal S., Sikandar A., Din S., Khan I., Ashraf S., Khan R.U., Tufarelli V., Laudadio V. (2021). Feeding of phytobiotics and exogenous protease in broilers: Comparative effect on nutrient digestibility, bone strength, and gut morphology. Agriculture.

[56] Farrokhi H., Abdullahpour R., Rezaeipour V. (2021). Influence of dietary phytase and protease, individually or in combination, on growth performance, intestinal morphology, microbiota composition, and nutrient utilization in broiler chickens fed sesame meal-based diets. Ital. J. Anim. Sci..

[57] Olukosi O.A., Beeson L.A., Englyst K., Romero L.F. (2015). Effects of exogenous proteases without or with carbohydrases on nutrient digestibility and disappearance of non-starch polysaccharides in broiler chickens. Poult. Sci..

[58] Kies A.K., De Jonge L.H., Kemme P.A., Jongbloed A.W. (2006). Interaction between protein, phytate, and microbial phytase: In vitro studies. J. Agric. Food Chem..

